# Virtual Reality Analgesia for Pediatric Dental Patients

**DOI:** 10.3389/fpsyg.2018.02265

**Published:** 2018-11-23

**Authors:** Barbara Atzori, Rosapia Lauro Grotto, Andrea Giugni, Massimo Calabrò, Wadee Alhalabi, Hunter G. Hoffman

**Affiliations:** ^1^Department of Health Sciences, Università degli Studi di Firenze, Florence, Italy; ^2^Multidisciplinary Analysis of Relationships in Health Care (M.A.R.H.C.) Joint Laboratory, Uniser and Università degli Studi di Firenze, Florence, Italy; ^3^Medical Practitioners and Dentists Board, Prato, Italy; ^4^Department of Computer Science, King Abdulaziz University, Jeddah, Saudi Arabia; ^5^Computer Science, Effat University, Jeddah, Saudi Arabia; ^6^Mechanical Engineering, University of Washington, Seattle, WA, United States

**Keywords:** virtual reality, pain, analgesia, attention, distraction, dental, dental caries, children

## Abstract

**Background:** Dental procedures often elicit pain and fear in pediatric dental patients.

**Aim:** To evaluate the feasibility and effectiveness of immersive virtual reality as an attention distraction analgesia technique for pain management in children and adolescents undergoing painful dental procedures.

**Design:** Using a within-subjects design, five patients (mean age 13.20 years old, SD 2.39) participated. Patients received tethered immersive interactive virtual reality distraction in an Oculus Rift VR helmet (experimental condition) during one dental procedure (a single dental filling or tooth extraction). On a different visit to the same dentist (e.g., 1 week later), each patient also received a comparable dental procedure during the control condition “treatment as usual” (treatment order randomized). After each procedure, children self-rated their “worst pain,” “pain unpleasantness,” “time spent thinking about pain,” “presence in VR,” “fun,” and “nausea” levels during the dental procedures, using graphic rating scales.

**Results:** Patients reported significantly lower “worst pain” and “pain unpleasantness,” and had significantly more fun during VR, compared to a comparable dental procedure with No VR. Using Oculus Rift VR goggles, patients reported a “strong sense of going inside the computer-generated world,” without side effects. The dentist preferred having the patients in VR.

**Conclusion:** Results of this pilot study provide preliminary evidence of the feasibility of using immersive, interactive VR to distract pediatric dental patients and increase fun of children during dental procedures.

## Introduction

### Traditional Analgesia

Pain during dental procedures is common, especially during invasive dental treatments such as tooth extractions or dental cavity fillings (Costa et al., 2012). Although local analgesics are routinely used to help control patients pain during dental procedures, pediatric patients often experience pain and anxiety during dental procedures (Guelman, 2005). Experiencing pain and anxiety during dental procedures can result in several negative consequences, such as higher levels of dental fear, uncooperative behaviors and a general dissatisfaction of the patient with dental care (Guelman, 2005). Unpleasant early dental/medical experiences can affect patients’ perception of healthcare, can increase pain and suffering during subsequent medical visits, and can reduce preventative healthcare, affecting lifelong health (El-Housseiny et al., 2014). El-Housseiny et al. (2014) recently conducted a study on dental fears in children. The children reported the following fears most prominently, ‘fear of usual dental procedures and injections,’ ‘fear of strangers’ (i.e., the dentist), ‘fear of general medical aspects of treatment,’ and ‘fear of health care personnel.’ Children with fear of dentist have more cavities/caries, and visit dentists less often, than children who do not have fear of dentist (Milsom et al., 2003). One study found that over half of children with dental fears became difficult to handle or exhibited problematic behaviors during the dental procedures (Goumans et al., 2004). It is recommended that children visit a dentist every six to 12 months, and for good reason. In one recent study of school children (aged 9–12 years) from a randomly selected sample of primary schools from Sharfia area of Jeddah Saudi Arabia, over 75% of the children had one or more carious first permanent molars (i.e., cavities/tooth decay). With regular visits to the dentist, cavities and more serious problems can often be prevented, but many children do not want to go to the dentist. Children’s learned aversion to visiting the dentist could be prevented by making dental visits less painful and more fun.

Ironically, when patients avoid going to the dentist, what could have been treated early as a tooth filling (preventative medicine), left untreated, may lead to advanced tooth decay such that patients require tooth extraction and/or root canal. Inflammation of the gums surrounding the infected tooth makes the dental care more painful, and healing after surgery takes longer with more advanced tooth decay. In some cases, unpleasant dental experiences can generalize to avoidance of healthcare in general.

Several techniques, both pharmacological and psychological, can be used to reduce patients’ pain and anxiety during dental procedures. Local anesthesia is the most frequent pharmacological technique to reduce dental pain. Ideally, local anesthesia results in complete absence of pain in the anesthetized area during dental procedures. However, local anesthesia requires an injection into the jaw with a long needle, and patients often refuse it because they consider the injection painful and or because patients fear/avoid needles (Kuscu and Akiuz, 2007). Among the psychological techniques for pain management, distraction is a simple psychological non-drug pain control technique that can be used in addition to traditional pain medications, to help control acute pain during medical procedures. According to the Attention Pain Theory by Eccleston and Crombez (1999), distraction can reduce the amount of attentional resources the patient’ brain has available to process incoming neural signals from pain receptors, with the result of a reduced subjective pain experience. However, the effectiveness of traditional distractions, such as music, for reducing pain and fear is often limited (Aitken et al., 2002; Koller and Goldman, 2012; Bellieni et al., 2013).

### Virtual Reality Analgesia via Attention Distraction

Virtual Reality (VR) analgesia is showing promise as an effective pain distraction technique for helping reduce the suffering and increasing the amount of fun children experience during painful medical procedures (Hoffman, 1998, 2004; Hoffman et al., 2006, 2011; Atzori et al., 2017).

The essence of immersive virtual reality is the user’s illusion of going inside the 3D computer generated world, as if the virtual world is a place the patient is visiting. Researchers propose the following explanation for why VR reduces pain (Hoffman, 1998; Hoffman et al., 2000, 2011). “Being there” in the virtual world, floods the brain with information. The brain is so pre-occupied with processing information presented via virtual reality, that the patient has less attention available to process incoming pain signals. VR allows the user to be immersed in a computer-generated environment. Patients wear a Head Mounted Display (HMD) that blocks the patients view of the real world, substituting computer generated visual images and sound effects. VR may also tap into a natural desire of patients to “escape” from painful situations. Among adult dental patients, preliminary studies have shown virtual reality was effective for helping reduce pain in patients undergoing periodontal scaling and root planning (Hoffman et al., 2001; Furman et al., 2009) and unspecified dental procedures (Wiederhold et al., 2014). A growing number of studies have shown the effectiveness of VR for reducing pain of severe burn patients, including children (see Hoffman et al., 2011 for a review), but the effectiveness of highly immersive, interactive Oculus Rift VR for reducing pediatric dental pain and increasing fun during dental procedures, is currently unknown.

The current pilot study is the first to explore the feasibility, acceptability and the effectiveness of immersive VR to reduce pain during dental procedures such as dental fillings, and to explore the dentist’s thoughts and opinions on the feasibility/applicability of this technique during dental procedures.

## Materials and Methods

### Experimental Subjects

For 6 months, patients aged 7–17 years, who needed dental fillings or a tooth extraction during two visits were recruited. Patients were selected in a Private Dental Practice in the city of Prato (ITALY) with the help of the staff assistant who schedules patients, according to the following criteria based on the existent literature (Atzori et al., 2017). To be included, children and adolescents had to be able to understand Italian language and complete the tests, and had to be able to wear the helmet and interact with the VR environment, without any physical or psychological impairments. Patients were excluded from the study if they needed other kinds of procedures during the same visit, if they had a diagnosis of epilepsy, if they were not accompanied by parents, and patients were excluded if they were older than 17 years or younger than 7 years old.

Five patients, three males (aged 11, 12, and 14 years old), and two females (aged 12 and 17 years) met the inclusion criteria and underwent tooth extraction or dental fillings on two dental visits separated by at least 1 week between visits.

### Procedure

The protocol used in the current study was approved by the IRB ethics committee at the University of Florence Italy. The study was undertaken with the understanding, approval and written consent of each subject and their parent/guardian. The protocol was conducted under internationally accepted ethical standards and was approved by the dentists of the Dental Private Practice. Children and adolescents meeting the inclusion and exclusion criteria were referred to the psychologist researcher. Selected patients and their parents were approached in the waiting room of the Dental Practice to determine if they had any interest in participating. Interested families accompanied the psychologist researcher into a private room where they were informed about what would be involved, and if they were interested, they signed written informed assent/consent forms. Each patient received VR during one dental procedure, and received no VR during a second comparable procedure on a different day (e.g., 1 week later). Using a within-subjects crossover design, with treatment order randomized, each patient received Yes VR on 1 day, and No VR on the second visit (or No VR on their first visit, and Yes VR on their second visit). No reward was given to patients for participating.

### Measures

Pain levels, the quality of the VR experience, nausea and fun were measured using the Italian translation of the 0–10 graphic rating scale (GRS, Tesler et al., 1991; Hoffman et al., 2014) questionnaire adopted to evaluate pain, the quality of VR experience, fun and nausea (Hoffman et al., 2006). The cognitive, affective and sensory components of pain were evaluated by asking patients to respond to the following questions with a score between 0 and 10: (1) “Rate your WORST PAIN during the most recent pain stimulus (pain intensity). 0 no pain at all, 1–4 mild pain, 5–6 moderate pain, 7–9 severe pain, 10 excruciating pain.” (2). How much TIME did you spend thinking about your pain during this most recent pain stimulus? (10-cm line with numeric and word descriptors beneath it: 0 = none of the time; 1–4 some of the time; 5 half of the time; 6–9 most of the time; and 10 all of the time). (3) How UNPLEASANT was the most recent pain stimulus? (10-cm line with numeric and word descriptors beneath it: 0 not unpleasant at all; 1–4 mildly unpleasant; 5–6 moderately unpleasant; 7–9 severely unpleasant; and 10 excruciatingly unpleasant). (4) How much FUN did you have during the most recent pain stimulus? (10-cm line with numeric and verbal descriptors: 0 no fun at all; 1–4 mildly fun; 5–6 moderately fun; 7–9 pretty fun; 10_extremely fun).

Patients were asked to respond to the following questions with a score between 0 and 10: While experiencing the virtual world, to what extent did you feel like you WENT INSIDE the virtual world? (10-cm line with numeric and verbal descriptors: 0 = I did not feel like I went inside at all; 1–4 mild sense of going inside; 5–6_moderate sense of going inside; 7–9 strong sense of going inside; 10 I went completely inside the virtual world). How REAL did the virtual objects seem to you during virtual reality? 0 = completely fake, 1–4 somewhat real, 5 = moderately real, 6–9 = very real, 10 = indistinguishable from a real object. To what extent (if at all) did you feel nausea (sick to your stomach) as a result of experiencing the virtual world during the most recent VR session? (from 0 = “no nausea at all,” 1–4 = mild nausea, 5 = moderate nausea, 7–9 = severe nausea, 10 = vomit).

The dentist’s experience during the procedure while the patients were using VR distraction, was investigated with a semi-structured interview of 30 min conducted by a psychologist at the end of data collecting (July 2016). The dentist answered the following questions: (1) How did you feel when you performed the procedure and the patient was interacting with VR, compared to the standard routine? (2) What do you think about patients’ experience during VR? (3) Did you find any impediment for the use of VR during dental procedures? and (4) Do you have any suggestion to improve VR distraction?

### Immersive Virtual Reality System

The current study used Oculus Rift DK2 and CV1 virtual reality goggles^1^, with two miniature computer screens, one screen per eye. The goggles received video and audio input from an MSI GT Series GT72 Dominator Pro G-1252 Gaming Laptop 6th Generation Intel Core i7 6700HQ (2.60 GHz) 16 GB Memory 1 TB HDD 512 GB SSD NVIDIA GeForce GTX 980M 4 GB GDDR5 17.3” with Windows 10 Home 64-Bit. Patients interacted with SnowWorld^2^, a virtual environment specifically designed for pain management of immobilized patients with severe burn injuries during painful procedures such as wound cleaning and range of motion exercises (Hoffman et al., 2001). In SnowWorld, patients have the illusion of going into an icy canyon where they throw snowballs at penguins, snowmen and other characters. The patient interacted with the virtual environment using a wireless mouse. SnowWorld VR software is specifically designed to be distracting, pleasant and non-nauseogenic, and to be used by patients who need to keep their heads and bodies still during the medical procedure (Hoffman et al., 2001). Traditional VR gaming software (which typically encourages head and body movements) could not be used by dental patients, who must remain very still during the dental procedures.

### Data Analysis

Within-subjects, paired *t*-tests were adopted to compare pain, nausea and fun levels between the “No VR” condition and the “Yes VR” condition. A researcher not involved in data collection carried out data analysis using the statistical Software SPSS 23. Results were considered significant when associated with *p*-values less than 0.05, using two tailed paired *t*-tests.

## Results

### Pain

Mean pain ratings were significantly lower during VR compared to the control condition for affective, and sensory components of pain. The mean “pain unpleasantness” during No VR was 2.40 (*SE* = 1.52), and dropped to 0.60 (*SD* = 0.55) during virtual reality, *t*(4) = 3.67, *p* < 0.05, *SD* = 1.10. The mean “worst pain” was 3.80 (*SD* = 2.59) during No VR, and this dropped to were 2.20 (*SD* = 1.79) during virtual reality, *t*(4) = 3.14, *p* < 0.05, *SD* = 1.14. One patient showed no reduction in pain during VR, the other four patients all reported reductions in pain during VR. Although the difference in “time spent thinking about pain” was not statistically significant for this measure, responses showed the predicted pattern of results. Patients spent more time thinking about their pain during No VR (mean = 2.60, *SD* = 1.95) vs. during Yes VR (mean = 1.00, *SD* = 1.00), *t*(4) = 2.36, *p* = 0.08 NS, *SD* = 1.52.

### Quality of IVR Experience, Fun and Nausea

While undergoing the painful dental procedure while interacting with VR, patients reported mean presence ratings of 7.40 (*SD* = 2.70) corresponding to “a strong sense of going inside the computer generated world,” and a mean of 7.40 (*SD* = 1.82) for the realism of VR objects, corresponding to “very real.” Mean nausea ratings were considered negligible in both conditions (<1 on a 0–10 scale). When interacting with VR, patients reported significantly higher levels of fun during the painful procedure, compared to the control condition. Fun during No VR (mean = 3.20, *SD* = 4.32) was “mildly fun” vs. “pretty fun” during Yes VR (mean = 8.20, *SD* = 2.49), *t*(4) = 2.80, *p* < 0.05, *SD* = 4.00.

### Dentist’s Experience

All patients had the same dentist. The dentist who performed all ten procedures (9 dental fillings and one tooth extraction) was also one of the authors of the current manuscript.

During a semi-structured interview after the study, the dentist made the following observations and comments. (1) The dentist felt more relaxed and was able to be more concentrated on his job when he performed the dental procedures while the patient was interacting with VR, compared to the routine standard care.

(2) The dentist considered patients less stressed during the interaction with VR and thought that they felt less pain than during the standard care. In the dentist’s opinion, the VR system was suitable both for children and adolescents, because all patients reported fun during the interaction with the virtual world and many of them wanted to continue playing after the end of the procedure. The dentist considered VR to be an effective distraction technique especially for patients with high levels of anxiety.

(3) No impediment or contraindication emerged. In the dentist’s opinion, the VR goggles/VR system didn’t impede the dentist’s ability to perform the dental procedures, and the dentist was able to easily communicate with the patient. The dentist found SnowWorld suitable for all patients.

(4) The dentist highlighted the need of a new VR software with a “hot” scenario in the future, because, he was concerned that the illusion of cold sensations could evoke pain during dental procedures in some patients (e.g., patients with cold sensitive teeth). Moreover, he expressed the desire to extend the use of VR pain management to also include adult patients.

## Discussion

The current pilot study was conducted as a proof of concept, to explore the feasibility of using a new generation of mass produced commercially available Virtual Reality to distract children during painful/fear inducing dental procedures. The current study tested the effects of immersive, interactive Oculus Rift virtual reality distraction as a psychological technique to control pain during tooth extraction and dental fillings/cavities in children and adolescents. Based on the Interruption of Attention Pain Model by Eccleston and Crombez, we predicted that patients focusing their attentional resources on the virtual environment, would experience less pain, including the cognitive, affective and sensory components of pain, and we predicted patients would report having more fun during their dental procedure, on the day they received VR compared to the day they received standard of care with No VR. During VR, patients reported a significant 42% reduction in their “worst pain ratings, a significant 75% reduction in patients ratings of “pain unpleasantness” and patients reported a significant 61% increase in their ratings of how much fun they experienced during the dental procedure. Despite having to keep their heads still, and using a computer mouse to look around and shoot snowballs at objects in the virtual world, patients reported a “strong sense of going inside the computer-generated world” during VR, without side effects.

Although promising, the current pilot study has several limitations. First of all, the dentist who declared the “Dentist’s experience” is one of the authors, raising a high risk of confirmation bias. The small sample size is another limitation. Studies with small samples sizes are vulnerable to the possibility that results may be biased if one single patient reports an extreme value overshadowing other patients’ response. Fortunately, this was not a problem in the current study. One patient showed no reduction in pain during VR, the other four patients all reported reductions in pain during VR, with no outliers. However, small sample size is always an important concern. Because it is not possible to make broad scientific conclusions based on the results of studies that use small sample sizes (Campbell and Stanley, 1963), the current study must be followed up with larger, more carefully controlled studies. Another limitation is that the patients only received VR during one visit. Moreover, because all patients used VR for the first time during the current research, results could possibly be due in part to a novelty effect. Future research is needed to determine whether virtual reality continues to reduce pain when used repeatedly, and ideally to compare immersive VR to other distraction using emerging technologies (e.g., augmented reality with see-through glasses).

The current study showed no problems with VR induced nausea. Having patients keep their heads still (crucial in the current study) greatly reduces the computational demands on the high performance gamers VR computer, reducing lag that can lead to VR simulator sickness/motion sickness in some people (e.g., for tips on using VR with children see^3^,^4^). The current results also provide preliminary evidence that VR can also be an effective technique to promote good emotions and help patients cope with painful procedures in a non-stressful manner. Indeed, patients that interacted with VR during tooth extraction and dental fillings reported significantly higher levels of fun, compared to No VR, treatment as usual. During an interview after completing the study the dentist who performed the procedures suggested that VR distraction could be especially effective for anxious patients. Future studies should evaluate VR effectiveness for pain management comparing dental fears patients vs. patients do who do not have dental fears. For many children, experiences at the dentist give the young pediatric patients their first impressions about visiting healthcare givers.

VR could also be used to distract patients during needle injections into their gums before dental procedures (Atzori et al., 2017). We predict more pediatric dental patients would be able to tolerate getting local anesthesia injections, if they are in virtual reality during the injection. In that case they could benefit from both local analgesia, and continue to use virtual reality during their dental procedure. The greatest total analgesia will likely be achieved when immersive interactive VR + traditional pain medications are used concurrently (Hoffman et al., 2007).

Future research is needed to determine whether immersive interactive VR has any long term benefits for improving children’s attitudes toward dental visits, and whether VR can improve children’s attitudes toward healthcare in general, and whether more positive experiences using VR during dental care increases patients future willingness to seek healthcare.

In conclusion, the present study supports the feasibility of VR as a distraction technique for pain management in children and adolescents. The results of this preliminary pilot study showed that this psychological technique can help reduce pain during tooth extraction and dental fillings without side effects, and made dental procedures more fun. Recent mass production of immersive VR goggles has increased the availability and affordability of Oculus Rift VR helmets, and there is growing interest in non-pharmacological techniques for pain management, making VR analgesia a promising direction for future research.

## Ethics Statement

This study was performed in accordance with the provisions of the Declaration of Helsinki. We obtained informed written consent from the patients and their caregivers. The patients’ anonymity has been preserved. The protocol was conducted under internationally accepted ethical standards and it was approved by the Department of Health Sciences (University of Florence) and by the Dentists of the Dental Practice.

## Author Contributions

All authors listed have made a substantial, direct and intellectual contribution to the work, and approved it for publication.

## Conflict of Interest Statement

The authors declare that the research was conducted in the absence of any commercial or financial relationships that could be construed as a potential conflict of interest.

## Footnotes

1 www.oculusvr.com

2 www.vrpain.com

3 www.commonsensemedia.org/research/virtual-reality-101

4 https://www.commonsensemedia.org/about-us/news/press-releases/common-sense-report-highlights-potential-impact-of-virtual-reality-on
